# Serum biomarkers for liver fibrosis assessment

**DOI:** 10.1515/almed-2023-0081

**Published:** 2023-11-14

**Authors:** Julia Maroto-García, Ana Moreno Álvarez, María P. Sanz de Pedro, Antonio Buño-Soto, Álvaro González

**Affiliations:** Biochemistry Department, Clínica Universidad de Navarra, Pamplona, Spain; Laboratory Medicine Department, Hospital Universitario La Paz, Madrid, Spain; Hospital La Paz Institute for Health Research (IdiPaz), Madrid, Spain; Navarra Institute for Health Research (IdiSNA), Pamplona, Spain

**Keywords:** liver fibrosis, liver fibrosis biomarkers, non-invasive biomarkers, serum biomarkers

## Abstract

Liver fibrosis is the result of chronic liver injury of different etiologies produced by an imbalance between the synthesis and degeneration of the extracellular matrix and dysregulation of physiological mechanisms. Liver has a high regenerative capacity in the early stage of chronic diseases so a prompt liver fibrosis detection is important. Consequently, an easy and economic tool that could identify patients with liver fibrosis at the initial stages is needed. To achieve this, many non-invasive serum direct, such as hyaluronic acid or metalloproteases, and indirect biomarkers have been proposed to evaluate liver fibrosis. Also, there have been developed formulas that combine these biomarkers, some of them also introduce clinical and/or demographic parameters, like FIB-4, non-alcoholic fatty liver disease fibrosis score (NFS), enhance liver fibrosis (ELF) or Hepamet fibrosis score (HFS). In this manuscript we critically reviewed different serum biomarkers and formulas for their utility in the diagnosis and progression of liver fibrosis.

## Introduction

Liver fibrosis is the result of chronic liver injury of different etiologies, including viral hepatitis, alcohol abuse, metabolic diseases such as non-alcoholic fatty liver disease (NAFLD) now known as metabolic dysfunction-associated steatotic liver disease (MASLD) [1], autoimmune diseases, and cholestasic liver diseases [2, 3]. It is produced by dysregulation of physiological remodeling mechanism, activation of myofibroblasts, and formation of a fibrous scar that may eventually lead to the development of cirrhosis [4]. The common feature in liver fibrosis pathologies represents an imbalance between the synthesis and degeneration of the extracellular matrix (ECM) that affects its structure and properties [5]. The liver has a high regenerative capacity; however, when the damage occurs persistently, this regeneration develops into chronic diseases, such as fibrosis, which is characterized by excess accumulation of ECM [6, 7]. Liver fibrosis can be reversible especially in the early stage [4] before cirrhosis and organ failure, so it is important to diagnose it as soon as possible to establish an adequate treatment. Whereas in advanced liver disease there is impaired liver regeneration in both experimental models and patients [8].

**Figure 1:**
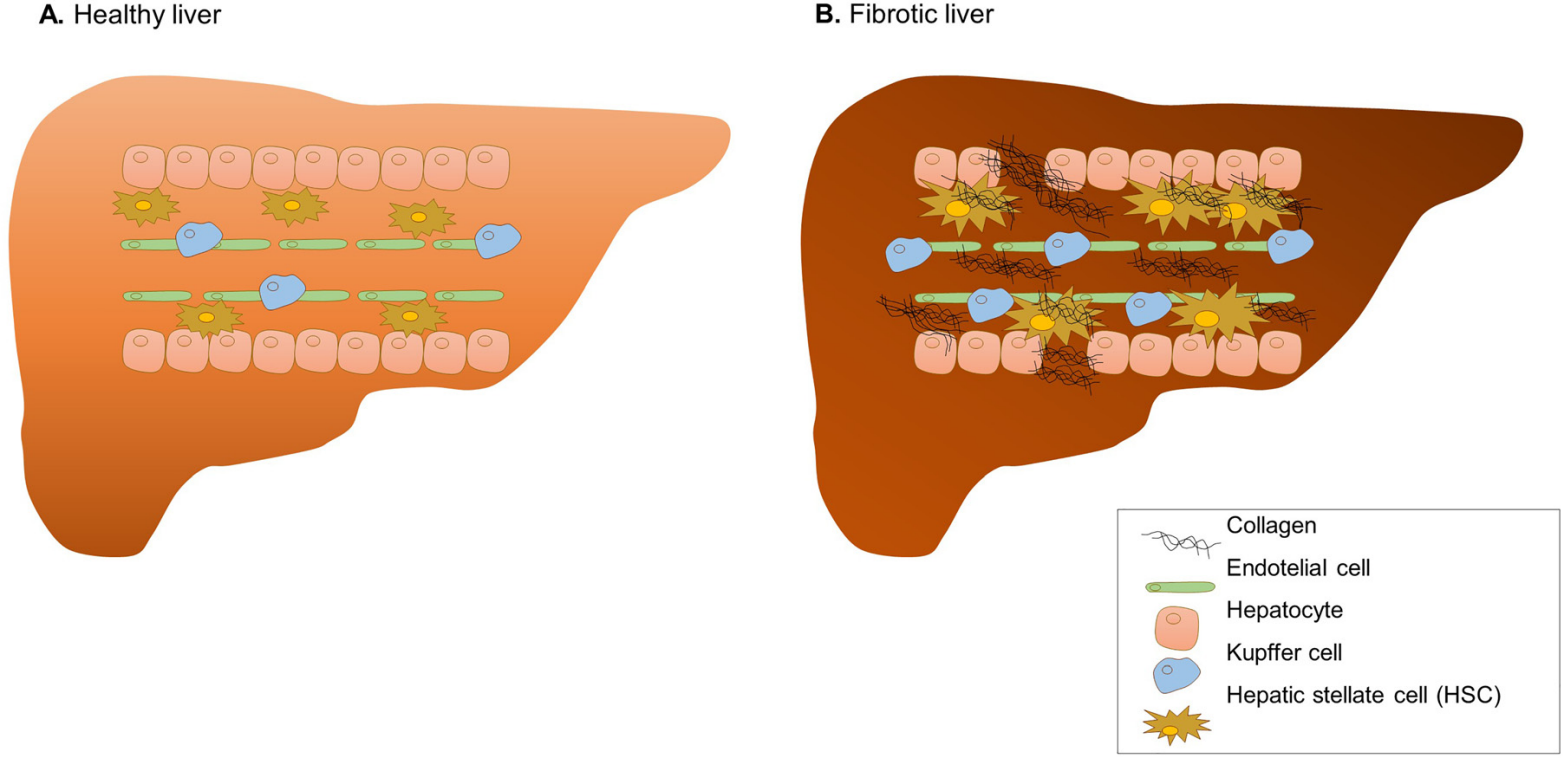
Differences between healthy and fibrotic liver.

Liver injury causes hepatocyte damage and disturbs tissue homeostasis, generally accompanied by inflammation [9]. When this situation occurs, it cause a pro-inflammatory response of Kupffer cells and an infiltration of immune cells that favor the activation of hepatic stellate cells (HSCs) into collagen-producing myofibroblasts [10, 11]. HSCs are the main controllers of ECM turnover, a process normally balance by anti-fibrotic mechanisms that inactivate myofibroblast or stimulate its apoptosis [10]. In chronic liver diseases activated myofibroblast conduce to a downregulation of matrix metalloproteinases (MMPs), upregulation of MMP-inhibitors (TIMPs), and secretion of wisteria floribunda agglutinin-positive Mac-2 binding protein (WFA+-M2BP) [12], which are implied in ECM degradation. MMPs are the main enzymes implicated in ECM degradation and TIMPs are capable of regulating the proteolytic activities of MMPs in tissues [13]. In addition, activated-HSC are the most important contributors to collagen deposition in the space of Disse, which results in gradual thickening of the space causing an increase in the portal pressure. Thus, excessive collagen accumulation occurs and the matrix regeneration fails, leading to an increase in liver stiffness [14] (Figure 1).

Currently, the gold standard for assessing the degree of liver fibrosis remains liver biopsy, using histopathological scoring systems such as METAVIR, the most widely used, which establishes four stages of liver fibrosis progression as follows: F0, no fibrosis; F1, mild fibrosis (portal fibrosis without septa); F2, moderate fibrosis (portal fibrosis and few septa); F3, advanced fibrosis (numerous septa without cirrhosis); and F4, cirrhosis [15]. Liver biopsy has well-known limitations including invasiveness, poor acceptability, sampling variability, cost, and inter-observer variation in interpretation [16]. The development and use of other non-invasive biomarkers that can be used in the diagnosis and progression of liver disease is therefore necessary.

Here we critically review liver fibrosis blood biomarkers considering direct biomarkers those derived directly from the ECM formation and degradation process or the molecular pathogenesis of fibrogenesis and fibrinolysis, while indirect biomarkers include biochemical parameters that reflect alterations in liver function and liver injury [17].

## Indirect biomarkers

### Liver enzymes

Alanine aminotransferase (ALT) and aspartate aminotransferase (AST) provide information about hepatocyte injury. Decreased levels of ALT [18] while increased AST and AST/ALT ratio are found in advanced liver disease patients [19]. Also, up to 80 % of MASLD patients have aminotransferase concentrations within the normal range, so they are not considered reliable and accurate predictors for its diagnosis [20]. Furthermore, it has been shown that patients with normal ALT levels and alterations in glucose metabolism and insulin resistance could develop MASLD; likewise, normal aminotransferases results are not consistent criterion to exclude patients for further studies, such as image techniques or liver biopsy [21]. Also AST/ALT ratio has been used to establish cirrhosis risk in patients with chronic viral hepatitis [22, 23], non-alcoholic steatohepatitis (NASH), alcoholic liver disease (ALD) [24, 25] and primary biliary cirrhosis (PBC) [26], but nowadays, this ratio cannot be used alone in predicting different fibrosis stages as it does not discriminate between moderate fibrosis or severe fibrosis [27, 28].

### AST to platelets ratio index

The AST to platelet ratio index (APRI), was initially evaluated to assess whether patients with HCV had liver fibrosis or not [25], [26], [27], [28], [29], and to differentiate between liver fibrosis stages and cirrhosis [30, 31]. A modified APRI (m-APRI) which incorporates age and serum albumin levels in the APRI formula has been proposed (Table 1) [32]. This m-APRI has been shown to improve the prediction of advanced fibrosis and cirrhosis in viral hepatitis [33].

**Table 1:** Scores and formulas used in liver fibrosis assessing and staging.

Score/index	Formula
APRI	AST (IU/L)/upper limit of normal AST value (IU/L)/platelet count (10^9^/L) × 100
m-APRI	Age (years) × (AST (IU/L)/upper limit of normal AST value (IU/L))/platelet count (10^9^/L) × 100
BARD score	AST/ALT > 0.8 2 points
	BMI > 28 1 point
	Diabetes diagnosis 1 point
Forns index	7.811 – (3.131 × ln (platelet count (10^9^/L))) + (0.781 × ln (GGT (IU/L))) + (3.467 × ln (age)) – (0.014 × (cholesterol (mg/dL)))
FIB−4	AST (IU/L) × age (years)/(platelet count (10^9^/L) × √(ALT (IU/L)))
NFS	−1.675 + (0.037 × age (year)) + (0.094 × BMI (kg/m^2^)) + (1.13 × IFG/diabetes (yes = 1, no = 2)) + (0.99 × AST/ALT ratio) – (0.013 × platelets (10^9^/L)) – (0.66 × albumin (g/dL))
FibroTest	(4.467 × log (α2-MG)) – (1.357 × log (haptoglobin)) + (1.017 × log (GGT)) + (0.0281 × age (year)) + (1.737 × log (total bilirubin)) – (1.184 × apoA1) + (0.301 × sex (male = 1, female = 0)) – 5.540
Fibrometer NAFLD	(0.4184 × glucose (mmol/L)) + (0.0701 × AST (UI/L)) + (0.0008 × ferritin (μg/L)) – (0.0102 × platelet (10^9^/L)) – (0.0260 × ALT (UI/L)) + (0.0459 × body weight (kg) + 0.0842 age (year)) + 11.6226
Hepascore	Y/(1 + Y)
	Where Y=exp (−4.185818 − (0.0249 × age) + (0.7464 × sex) + (1.0039 × α2-MG) + (0.0302 × HA) + (0.0691 × total bilirubin) – (0.0012 × GGT))
HFS	1/(1 + e ^[5.390 − 0.986 × age [45–64 years of age] − 1.719 × age [≥65 years of age] + 0.875 × male sex − 0.896 × AST [35–69 IU/L] − 2.126 × AST [≥70 IU/L] − 0.027 × albumin [4–4.49 g/dL] − 0.897 × albumin [<4 g/dL] − 0.899 × HOMA−R ^ ^[2 − 3.99 with no diabetes mellitus] − 1.497 × HOMA−R [≥4 with no diabetes mellitus] − 2.184 × diabetes mellitus − 0.882 × platelets × 1.000/μL [155−219] − 2.233 × platelets × 1.000/μL [<155^^]]^).
Benlloch index	1/1 + e^−12.698 + (0.097 × (albumin/total protein ratio)) – (1.356 × (prothrombin time)) – (0.004 × (AST)) – (0.02 × (time since liver transplantation))^
ADAPT score	exp (log_10_ ((age × PRO-C3)/√platelet count) + diabetes (0 = absent; 1 = present))
ELF	2.494 + 0.846 ln (HA) + 0.735 ln (PIIINP) + 0.391 ln (TIMP-1)
CHI3L1 model	0.032 × AST − 0.012 × platelets + 0.012 × HA + 0.846 × log 10 (CHI3L1) − 4.752
M2BPGi COI	([M2BPGi]_sample_ − [M2BPGi]_negative control_)/([M2BPGi]_positive control_ − [M2BPGi]_negative control_)

α2-MG, alpha 2 macroglobulin; ALT, alanine aminotransferase; ApoA1, apolipoprotein A1; APRI, AST to platelet ratio index; AST, aspartate aminotransferase; BMI, body mass index; CHI3L1, Chitinase 3-like protein 1; ELF, enhanced liver fibrosis; GGT, gamma-glutamyltranspeptidase; HA, hyaluronic acid; HFS, hepamet fibrosis score; HOMA-R, homeostatic model assessment of insulin resistance; m-APRI, modified APRI; M2BPGi, Mac-2 binding protein glycosylation isomer; M2BPGi COI, Mac-2 binding protein glycosylation isomer cut-off index; NFS, non alcoholic fatty liver disease fibrosis score; IFG, impaired fasting glucose; PIIINP, propeptide of type III procollagen; TIMP-1, matrix metalloproteinases inhibitor type 1.

### BARD

This score was proposed by Harrison et al., taking into consideration the presence of type 2 diabetes mellitus, the patient’s body mass index (BMI) and liver serum enzymes activity using the AST/ALT ratio [34]. BARD score has a high negative predictive value (NPV) of around 96 % in MASLD patients [34]. Recently Park et al. [35] have reported in patients with MASLD the association between advanced liver fibrosis assessed by the BARD score, and an increased risk for cardiovascular disease (CVD) and mortality suggesting its relation with myocardial inflammation and ischemic stroke [35].

### Forns index

Forns index is a score system which combines age, gamma-glutamyltranspeptidase (GGT), cholesterol, and platelet count that has proved to be useful to identify patients without moderate hepatic fibrosis in chronic hepatitis C population [36]. Also, Forns index has been validated in biopsy proven MASLD patients in chronic liver disease population without decompensated cirrhosis or hepatocellular carcinoma [37]. Moreover, Romero et al. [38] described that in patients with genotype 1 CHC Forns index used in combination with APRI, shows a 95.2 % accuracy in predicting moderate fibrosis and a 91.7 % accuracy in detecting advanced fibrosis. Thus, Forns index has been shown as an accurate predictor of morbidities and mortality in MASLD patients, similar to APRI [39].

### FIB-4

FIB-4 is an index based on age, AST, ALT serum activities and platelet concentration (Table 1). It is probably the most widely used serum index for screening hepatic fibrosis as a first step, and its use is well recommended in MASLD clinical guidelines such as the American Association for the Study of Liver Diseases (AASLD) Practice Guidance on the Clinical Assessment and Management of Nonalcoholic Fatty Liver Disease [1, 40]. The most accepted FIB-4 cut-off for advanced fibrosis is 2.67 [41] but some studies have established it in 3.25 [42] (Table 2). Itakura et al. [43] found that FIB-4 has an accuracy rate of around 71 % in the diagnosis of cirrhosis due to HBV infection, and around 75 % in patients with HCV infection. Although different meta-analyses also found that FIB-4 as well as APRI were moderately effective for the assessment of fibrosis stage in chronic hepatitis B [44], [45], [46], FIB-4 has a higher diagnostic accuracy when compared with APRI for predicting moderate or advanced fibrosis and cirrhosis diagnosis [46]. As reported in different studies, FIB-4 has a high diagnostic value to assess cirrhosis, and moderate or severe fibrosis [47], [48], [49], [50], [51], [52], [53], [54]. FIB-4 is a useful tool in liver fibrosis screening because of its practicability and high NPV [55]. Thus, a cut-off of 1.3 has been proposed to discard advanced liver fibrosis [56]. However, the specificity for advanced fibrosis in patients aged ≥65 years is lower, resulting in a high false positive rate, so the proposed FIB-4 cut-off in this group of age increase to 2 [57]. Although the usefulness of this biomarker to rule out advanced liver fibrosis, in patients with a high prevalence such as diabetics, MASLD cannot be ruled out with a single FIB-4, if there is high suspicion, re-evaluation, or use of other more specific methods, is recommended [58, 59].

**Table 2:** Liver fibrosis serum biomarkers cut-off in literature.

Score/index	Patients/cohort	Diagnosis	Predict/rule out	AUC	Cut-off	Sensitivity, %	Specificity, %	PPV, %	NPV, %	References
AST/ALT	MASLD	Advanced fibrosis	Predict	0.83 (0.74–0.91)	>0.8	74	78	44	93	[60]
APRI	Patients with chronic viral hepatitis	Significant fibrosis	Predict	0.72 (0.69–0.75)	>0.5	79.9	48.4	67.3	64.4	[61]
	MASLD	Advanced fibrosis	Predict	0.67 (0.54–0.8)	>1	27	89	37	84	[60]
		Cirrhosis	Predict	0.77 (0.73–0.81)	>2	45.2	88.4	38.7	90.9	[61]
MAPRI	MASLD	Advanced fibrosis	Predict	0.84 (0.78–0.89)	>5.84	77.8	79.8	75.7	81.6	[32]
		Cirrhosis	Predict	0.83 (0.74–0.87)	>9	67.3	85.7	67.3	85.7	[32]
BARD	MASLD	Advanced fibrosis	Predict	0.77 (0.68–0.87)	>2	89	44	27	95	[60]
FORNS INDEX	ALD	Advanced fibrosis	Predict	0.83 (0.78–0.89)	>6.9	67	89	55	93	[62]
FIB-4	MASLD (age < 65)	Advanced fibrosis	Rule out	0.86 (0.78–0.94)	≤1.3	85	65	36	95	[42, 60]
			Predict		>3.25	26	98	75	85	[60]
	MASLD (age > 65)	Advanced fibrosis	Predict	NR	>2	77	70	12	98	[57]
	HIV/HCV-coinfected patients	Advanced fibrosis	Rule out	0.737	<1.45	66.7	71.2	38	89	[47]
			Predict		>3.25	26	96.6	64.5	82.6	[47]
		Advanced fibrosis	Rule out	0.802 (0.758–0.847)	<1.3	74	71	43	90	[41]
			Predict		>2.67	33	98	80	83	[41]
NSF	MASLD	Advanced fibrosis	Rule out	0.81 (0.71–0.91)	<−1.455	78	58	30	92	[60]
			Predict		>0.676	33	98	79	86	[60]
		Advanced fibrosis	Rule out	0.84 (0.81–0.88)	<−1.455	77	71	52	88	[63]
			Predict		>0.676	43	96	82	80	[63]
Fibrotest	Patients with chronic viral hepatitis	Significant fibrosis	Predict	0.78 (0.75–0.81)	>0.48	67.4	75.3	78.7	63.1	[61]
	Patients with chronic viral hepatitis	Cirrhosis	Predict	0.82 (0.79–0.85)	>0.74	62.6	84.4	40.1	93.1	[61]
Gp73	Chronic HBV patients	Significant liver inflammation	Predict	0.806 (0.748–0.856)	>85.7 ng/mL	43.59	97.18	89.5	75.8	[64]
	Chronic HBV patients	Significant fibrosis	Predict	0.742 (0.679–0.799)	>84.49 ng/mL	30.70	96.23	89.74	56.35	[64]
HFS	MASLD	Advanced fibrosis	Rule out	NR	<0.12	74.6	75.5	49.8	90.1	[65]
			Predict	NR	≥0.47	34.6	96.7	77.2	81.9	[65]
Benlloch index	Chronic HCV transplant patients	Significant fibrosis	Rule out	0.84	≤0.2	87	71	49	95	[66]
		Significant fibrosis	Predict	0.84	≥0.8	17	99	80	79	[66]
HA	Chronic liver diseases	Advanced fibrosis	Predict	NR	>90 μg/L	80.4	70.2	86.7	59.8	[67]
	Chronic liver diseases	Cirrhosis	Predict	NR	>210 μg/L	96.2	85.3	65.4	98.8	[67]
PCIIINP	Chronic liver diseases	Advanced fibrosis	Predict	NR	>90 μg/L	82	60.8	83.5	58.4	[67]
	Chronic liver diseases	Cirrhosis	Predict	NR	>150 μg/L	76.4	68.7	40.4	91.3	[67]
CIV	Chronic liver diseases	Advanced fibrosis	Predict	NR	>75 μg/L	63.1	83.8	90.4	48.4	[67]
	Chronic liver diseases	Cirrhosis	Predict	NR	>90 μg/L	80	75.8	47.8	93.2	[67]
PRO-c3	ALD	Advanced fibrosis	Predict	0.85 (0.79–0.90)	>15.6	81	73	38	95	[62]
	Chronic HCV patients	Advanced fibrosis	Predict	0.72 (0.65–0.78)	>20.2	71.4	71.9	NR	NR	[68]
ADAPT score	ALD	Advanced fibrosis	Predict	0.88 (0.83–0.93)	>6.328	86	78	44	97	[62]
	MASLD	Advanced fibrosis	Predict	0.86 (0.79–0.91)	>6.328	NR	NR	48.4	96.6	[69]
CHI3L1 OR YKL-40	MASLD	Advanced fibrosis	Predict	0.764	>165 μg/L	70	76.8	NR	NR	[70]
	ALD	Advanced fibrosis	Predict	NR	>330 μg/L	88.5	50.8	NR	NR	[71]
	HBV	Advanced fibrosis	Predict	0.97	>68.75 μg/L	95.2	89.7	NR	NR	[72]
	HCV	Advanced fibrosis	Predict	0.809	>186.4 μg/L	78	81	NR	NR	[73]
M2BPGi COI	HBV	Significant fibrosis	Predict	0.653 (0.608–0.698)	>0.25	74.8	47.3	NR	NR	[74]
	HBV	Advanced fibrosis	Predict	0.59 (0.50–0.67)	≥3.0	18.8	98.5	NR	NR	[75]
			Predict	0.795 (0.743–0.848)	>0.45	69.6	74.1	NR	NR	[74]
	HBV	Cirrhosis	Predict	0.914 (0.815–1)	>0.96	83.3	92.7	NR	NR	[74]
ELF	Chronic liver diseases	Severe fibrosis	Predict	0.86 (0.83–0.89)	≥10.48	62	89	73	83	[76]
	Liver fibrosis (EUROGOLF cohort)	Mild fibrosis	Predict	NR	>7.7	85	38	NR	NR	[77]
	Liver fibrosis (EUROGOLF cohort)	Advanced fibrosis	Predict	NR	>9.8	65	90	NR	NR	[77]
	Liver fibrosis (EUROGOLF cohort)	Cirrhosis	Predict	NR	≥11.3	38	97	NR	NR	[77]
	HBV	Advance fibrosis	Predict	NR	>9.8	62	66	55	72	[78]
Hepascore	HCV	Significant fibrosis	Predict	0.81	≥0.55	82	65	70	78	[79]
	Patients with chronic viral hepatitis	Significant fibrosis	Predict	0.78 (0.75–0.80)	>0.5	52.9	86.3	83.7	57.9	[61]
	HCV	Significant fibrosis	Predict	0.852 (0.778–0.926)	>0.5	67	92	NR	NR	[80]
	HCV	Advanced fibrosis	Predict	0.957 (0.918–0.995)	>0.5	95	81	NR	NR	[80]
	HCV	Cirrhosis	Predict	0.938 (0.872–1.000)	>0.84	71	89	NR	NR	[80]
	Patients with chronic viral hepatitis	Cirrhosis	Predict	0.86 (0.83–0.88)	>0.84	59	87.4	43.2	92.9	[61]
Fibrometers	Patients with chronic viral hepatitis	Significant fibrosis	Predict	0.79 (0.76–0.81)	>0.411	83.1	57.1	72	71.8	[61]
	Patients with chronic viral hepatitis	Cirrhosis	Predict	0.86 (0.83–0.89)	>0.442	43.9	95	58.1	91.5	[61]

ALT, alanine aminotransferase; APRI, AST to platelet ratio index; AST, aspartate aminotransferase; AUC, area under the curve; CHI3L1, chitinase 3-like protein 1; ELF, enhanced liver fibrosis; Gp73, Golgi protein 73; HA, hyaluronic acid; HFS, hepamet fibrosis score; HBV, hepatitis B virus; HCV, hepatitis C virus; M2BPGi COI, Mac-2 binding protein glycosylation isomer cut-off index; NAFLD, non-alcoholic fatty liver disease; NFS, NAFLD fibrosis score; NPV, negative predictive value; NR, not reported; OELF, original ELF; PIIINP, N-terminal propeptide of procollagen type III; PPV, positive predictive value.

### NAFLD fibrosis score

NAFLD fibrosis score (NFS) considers the same parameters as FIB-4 plus BMI, diabetes (yes/no) and albumin (Table 1). Its use has been recommended by the EASL-EASD-EASO Clinical Practice Guidelines [81] for MASLD patients to whether diagnose advanced liver fibrosis or not, as it has an area under the receiving operating characteristics curve (AUROC) >0.8, similar to FIB-4 (Table 2) [60, 63]. However, despite their practicability FIB-4 and NFS are still considered suboptimal as they have a substantial proportion of false-positive and false negatives when applied to the general population, thereby they should only be used in at-risk populations [82].

### Fibrotest

FibroTest™ (Biopredictive Paris), is a multimarker panel consisting of serum α2-macroglobulin, apolipoprotein A1, haptoglobin, total bilirubin, and GGT, adjusted for age and gender [83] (Table 1). Fibrotest has been validated in common liver diseases such as chronic hepatitis B (CHB) [84], Alcoholic liver disease (ALD) [85], and MASLD [83] for stratifying liver fibrosis. In a recent meta-analysis Vali et al. [86] concluded that Fibrotest has acceptable performance in detecting cirrhosis (AUC = 0.92) in MASLD patients, however, it showed limited accuracy in predicting moderate and advanced fibrosis (AUROC = 0.77 for both conditions). Similar results were found in chronic viral hepatitis patients by Degos et al. [61] (Table 2). Despite all, Fibrotest has good predictive values for diagnosing liver fibrosis in MASLD patients and thus it is included in EASL-EASD-EASO Clinical Practice Guidelines [81], also for the survival without liver-related deaths, the CVD-related deaths and the overall survival [87].

### Golgi protein 73 (Gp73)

GP73 is a transmembrane protein released by damaged cells, increased in the serum of chronic liver patients [88–90]. It is known to be highly expressed in patients with liver cirrhosis [90], and thus, it has shown great diagnostic value for liver cirrhosis [91, 92]. Recently, it has been demonstrated that in patients with compensated cirrhosis higher levels of GP73 are related to worse outcomes such as decompensation, hepatocarcinoma development, and liver-related deaths [93, 94]. Its use also has been validated in MASLD [95], ALD and viral hepatitis patients [64, 88, 96], proving to be a useful tool in the diagnosis of advanced fibrosis and cirrhosis, even better than FIB-4 or APRI [97].

### Hepamet fibrosis score (HFS)

HFS is a recently proposed new score validated in a large, multicenter European population of Caucasian ethnicity with biopsy-proven MASLD that includes age, sex, diabetes, Homeostatic Model Assessment of Insulin Resistance (HOMA-IR), AST, albumin, and platelets in its formula [65]. Its developers have reported that HFS identified patients with advanced fibrosis with greater accuracy compared to FIB-4 and NFS index. Although it is a novel score, several studies had validated its thresholds [98–100] and verified that HFS had the highest diagnostic accuracy and the highest negative predictive value when compared to NFS and FIB-4 in metabolic hepatic steatosis patients [101]. Moreover, HFS is as reliable as NFS, and FIB-4 for predicting cirrhosis, long-term liver-related events, hepatocarcinoma, and overall mortality, with higher performance in the prediction of moderate and severe fibrosis, explained by the fact of including the presence of diabetes in its formula or in non-diabetics patients, the HOMA-IR [102]. Moreover, higher levels of HFS (cut-off 0.12) are related to an increased risk of developing type 2 diabetes mellitus and arterial hypertension in MASLD patients, but NFS or FIB-4 could not predict this outcome [103].

### OWLiver

The patented metabolomic test OWLiver^®^ (One Way Liver S.L., Bilbao, Spain) is a fasting blood test able to measure the degree of MASLD development by measuring a panel of triacylglycerols serum biomarkers and BMI, which represents the fat and inflammation of the liver [104]. The triacylglycerols are measured by high-performance liquid chromatography and mass spectrometry (UHPLC-MS) and then all the results are taken together in an algorithm that gives the final OWLiver^®^ score [105]. Owliver is able to distinguish between normal liver and MASLD liver fibrosis showing an AUROC of 0.90 with a high sensitivity, which means a low rate of false negatives cases [106]. This score can also differentiate between simple steatosis and steatohepatitis pathology as reported in the prospective validation study, where patients had previously been diagnosed by liver biopsy [104]. When compared with liver biopsy, the OWLiver^®^ Care and OWLiver^®^ tests had a suboptimal performance in patients with type 2 diabetes mellitus patients, as reported by Bril et al. [107]. A lack of comparative studies between OWLiver^®^ and other non-invasive liver fibrosis scores, and the complexity of its methodology makes it difficult to be implemented in clinical practice.

### Benlloch index

Benlloch index is a model created to evaluate chronic HCV patients who have undertaken a liver transplantation. The aim of Benlloch index is to evaluate whether or not is necessary to start antiviral therapy and follow up carefully this group of patients in order to indicate retransplantation [66]. This index uses 4 indirect biomarkers AST, prothrombine time, albumin/total protein ratio, and time since liver transplantation (Table 1). It has demonstrated efficacy compared to liver biopsy in chronic HCV patients who have undertaken a liver transplantation showing an acceptable discriminative power differentiating significant and advance fibrosis [66].

## Direct biomarkers

### Extracellular matrix-deriving proteins

#### Hyaluronic acid

Hyaluronic acid (HA) is an important constituent of the extracellular matrix and is highly present in the liver [108]. Several cell types can secrete HA, the synovial lining cells and the HSC are responsible of its synthesis in the liver, while sinusoidal endothelial cells are involved in its degradation [109]. HA serum concentrations are elevated in liver diseases associated with fibrosis such as ALD [109, 110], MASLD [111–113], HCV [114–116], HBV [108, 117] and HIV-HCV coinfection [118, 119]. Therefore, it can be used as a non-invasive biomarker to assess the presence of liver fibrosis and to monitor disease progression [109, 120].

#### N-terminal propeptide of procollagen type III

N-terminal propeptide of procollagen type III (PCIIINP) is one of the major components of connective tissue that has been recently reported to perform well in the detection of fibrosis in type 2 diabetes patients [121]. Serum levels PCIIINP are increased in ALD or HCV patients and it correlates with the stage of liver fibrosis [122–124]. In addition, PCIIINP plasma levels have been studied and showed to perform better than APRI or FIB-4 as a non-invasive biomarker for diagnosing liver fibrosis stage in children and adolescents with biopsy-proven MASLD [125].

#### Laminin and type IV collagen

Type IV collagen (CIV) and laminin (LN), have been widely evaluated in ALD, viral hepatitis, and MASLD patients [111, 126]. In addition, serum CIV and LN levels correlated with the fibrotic stage in HCV patients and were reported as accurate non-invasive markers of liver fibrosis and liver inflammation [127]. Otherwise, LN has a lower diagnostic performance compared to HA and CIV [128].

Serum HA, PCIIINP and CIV have been proposed as biomarkers for an accurate diagnosis of liver fibrosis in different chronic liver diseases, furthermore, HA is the best for screening liver cirrhosis [67]. However, Stefano et al. reported that CIV could predict the presence of moderate and advanced fibrosis in MASLD patients with a better AUROC than LN, HA, and PCIIINP [129].

#### N-protease cleavage site of PIIINP

N-protease cleavage site of PIIINP (PRO‐C3) is a systemic marker of type III collagen formation and fibroblast activity. Thus, is therefore directly related to the development of liver fibrosis. It has been proved to detect liver fibrosis, progression rate and treatment response in patients with chronic liver disease [68, 130–132]. PRO-C3 was firstly proved to differentiate moderate from severe fibrosis in CHC patients and, also identify CHC patients with fibrosis progression more accurately than the common used FibroTest [68]. Moreover, it has been found that in ALD and MASLD population the utility of PRO-C3 increases when used in an algorithm known as the ADAPT score, which includes age, diabetes and platelets count in its formula (Table 1) [62, 69]. The ADAPT score shows superiority when compared to APRI, FIB-4 and NFS, and also has the advantage of being able to stratify fibrosis or cirrhosis in contrast to the other non-invasive biomarkers, which only allow dichotomous results [69].

#### Matrix metalloproteinases (MMPs) and tissue inhibitor of metalloproteinase (TIMPs)

TIMP-1 was the only metalloproteinase that could be considered an independent predictor of histological fibrosis as reported in a study conducted on MASLD patients [133]. However, Livzan et al. reported TIMP-2 as a potential non-invasive marker for the diagnosis of liver fibrosis in patients with MASLD having a good correlation with the severity of fibrosis [134]. Also, Boeker et al., reported that MMP-2 can be used to detect cirrhosis with high efficacy in patients with chronic HCV, and that performed better than HA or TIMP-1 [135]. Furthermore, the MMP-2/TIMP-1 ratio was proposed as an indicator of Interferon-γ treatment response in CHC patients, describing a greater decrease in ratio levels in responders compared to no-responders or not treated patients [136]. In addition, Munsterman et al. described higher levels of TIMP-1 and TIMP-2 in severe fibrosis than in mild or no fibrosis in MASLD patients, and also that MMP-9 was the only ECM component correlated with inflammation severity [137]. Moreover, MMP-7 has been recently described as an independently associated liver fibrosis biomarker capable of improving the diagnostic performance in older MASLD patients when combined with the enhanced liver fibrosis (ELF) test [138].

#### Chitinase 3-like protein 1 (CHI3L1)

CHI3L1 also called YKL-40, is a protein secreted by macrophages, neutrophils, vascular smooth muscle cells, cancer cells, etc. but its expression in the liver is higher than in other tissues. CHI3L1 has several functions as promoting extracellular matrix degradation and tissue remodeling [139]. CHI3L1 levels have been related to the staging of liver fibrosis in MASLD [70], ALD [71], HBV [72] and HCV patients [73]. Huang et al. showed that CHI3L1 is a good marker in differentiating substantial fibrosis (AUC = 0.94), and advanced fibrosis (AUC = 0.96). Also, that CHI3L1 is superior to HA, PCIIINP, LN, and CIV for this purpose [140]. As reported by Saitou et al. [73], serum CHI3L1 levels outperform other non-invasive fibrosis biomarkers including CIV, HA, and PCIIINP for distinguishing advanced fibrosis from mild fibrosis with an AUC of 0.809 in HCV infection patients and that its levels decrease after therapy. Furthermore, a CHI3L1 model has been proposed and it was found to be superior to APRI and FIB-4 in predicting moderate fibrosis in HBV patients with ALT less than two times the upper limit of the normal range [141].

#### Mac-2 binding protein glycosylation isomer (M2BPGi)

M2BPGi is a glycoprotein produced by HSC, which functions as a messenger between HSC and Kupffer cells promoting fibrogenesis [142]. Thus, it has been recommended as an accurate biomarker for staging hepatic fibrosis [143, 144]. M2BPGi levels are expressed as a cut-off index (COI) in literature, and its usefulness in liver fibrosis has been validated in several studies in patients with different aetiologies such as HVC [145], HVB [75, 146] (Table 2), autoimmune hepatitis [147], NASH [148], MASLD [111, 149], biliary atresia [150], primary biliary cirrhosis [151], primary sclerosing cholangitis [152] and mortality in liver cirrhosis [153]. In a study in HBV patients, M2PBGi correlated with the fibrosis stage (F0-F4) and was superior to PLT count, HA, PCIIINP, TIMP-1, FIB-4 index, APRI, and ELF score for staging moderate fibrosis [154]. Similar results were found when compared to AST/ALT ratio, APRI and FIB-4 for detecting advanced liver fibrosis [74]. Furthermore, recent studies described M2BPGi as a biomarker in the follow-up after antiviral therapy in liver fibrosis patients [155–159], patients with high M2BPGi levels after antiviral treatment, must be followed up carefully for hepatocarcinoma development [155, 160]. Moreover, as reported in a study of HCV patients, M2BPGi was better than FIB-4 to distinguish different fibrosis stages after treatment with direct-acting antivirals [161].

Although the utility demonstrated of these direct biomarkers, they show better sensitivity and specificity when used combined [124], that is in algorithms or scores such as ELF, Hepascore or Fibrometer which will be described below.

### Calculated formulas or index using extracellular matrix deriving proteins

#### Enhance liver fibrosis (ELF)

ELF is a patented blood test (Siemens Healthineers, Erlagen, Germany) that measures three molecules involved in liver matrix metabolism (TIMP-1, PIIINP and HA) to give a score reflecting the severity of liver fibrosis. Since its appearance as the original ELF (OELF), the algorithm has suffered some modifications such as eliminating the parameter age, thus different thresholds have been reported [162, 163]. ELF has revealed good accuracy in predicting liver fibrosis [77, 164], having been validated in different chronic liver diseases such as ALD [165], MASLD [166], primary biliary cirrhosis [167] and viral hepatitis infection [78, 168, 169]. It is capable of distinguishing severe fibrosis, moderate fibrosis, and no fibrosis, with an AUC of 0.90, 0.82, and 0.76 respectively [170] (Table 2). Moreover, ELF as APRI and FIB-4 have been described to be useful in early identification of patients at high risk of severe post liver transplant hepatitis C recurrence [171]. Higher ELF levels have been related to worse clinical outcomes in patients with chronic liver disease, suggesting that could be used in prognostic [76]. The AASLD Practice guidance on the clinical assessment and management of MASLD, recommends its use as a second line specific test, being comparable to FibroScan in advanced liver fibrosis assessment [1, 172]. However, influence factors such as gender, age and time in which the blood test is conducted need to be taken into account when interpreting results to minimize the variability of the test [173].

#### Hepascore

Hepascore was first validated in predicting different stages of fibrosis among HCV infected patients. It combines socio-demographic variables like age and gender with blood-based parameters including bilirubin, gamma-glutamyl transferase, HA, and α2-macroglobulin [80] (Table 1). Nowadays is a widely used algorithm to detect moderate fibrosis in many chronic liver diseases [79, 174, 175]. As reported by Huang et al. [174], Hepascore had a better diagnostic ability for moderate and advanced fibrosis in CHC, CHB and ALD than MASLD and HIV co-infected viral hepatitis. Furthermore, it has been compared to other scores, and it showed significantly higher diagnostic values in ALD patients than APRI, and FIB-4 scores, however, it was similar compared to FibroTest, FibrometerA [85]. Contrarily, Chrostek et al. [176], revealed that Hepascore had a lower diagnostic value in a randomized group of alcoholic patients than APRI, Forns and FIB-4 when using Fibrotest as a matrix for comparing their diagnostic values. On the other hand, in patients with MASLD, is capable to identify advanced liver fibrosis [177]. In addition, Hepascore has been shown to predict accurately long-term risks such as decompensation, and hepatocarcinoma, both liver-related death in patients with metabolic dysfunction associated with MASLD [178]. However, large biological within-individual variations in non-fasting plasma HA are found in both health and chronic liver disease, thus the Hepascore system should be evaluated with caution in single measurement or clinical follow-up [179].

#### FibroMeters

FibroMeters is a family of patented blood biomarkers panels with several specificities, flexible to be adapted according to the cause of the chronic liver disease [180]. They included direct blood biomarkers such as HA, α2-macroglobulin, and indirect blood test prothrombin time, platelets, AST, ALT, GGT, bilirubin, urea, ferritin and other data such as age, body weight and gender (Table 1). FibroMeters were first developed and validated for the detection of fibrosis stage in patients with CHB or chronic hepatitis C (CHC) [181] and MASLD [182]. FibroMeter test for fibrosis staging in CHC showed an AUROC significantly higher than Fibrotest, Hepascore, APRI and FIB-4, as reported by Calès et al. [180]. In a meta-analysis in CHC patients, FibroMeter demonstrated superiority over both FibroTest and Hepascore in terms of overall diagnostic performance [183]. The standard FibroMeter was expanded to improve the diagnostic performance for cirrhosis, which uses specific coefficients with the same clinical and blood parameters as the standard FibroMeter, resulting in a PPV of 100 % in HCV patients [184]. Moreover, in MASLD patients FibroMeter has shown higher accuracy for moderate fibrosis than APRI or NFS [185]. FibroMeter virus second (V2G) and third generation (V3G) are both two tests that were initially developed for the diagnosis of moderate fibrosis in patients with hepatitis C [186], but in recent years they have also been validated in patients with MASLD [182, 187]. The latest version is the FibroMeter vibration-controlled transient elastography (VCTE), which is a combination of FibroMeter V3G and TE [188]. It has shown the best diagnostic accuracy in detecting advanced fibrosis when compared to Fibrometer VG2 and Fibrometer MASLD [189] and also is better than NFS, and TE for diagnosing severe liver fibrosis in MASLD patients [190]. Guillaume et al. [187], reported equal accuracy for ELF and FibroMeterV2G in patients with MASLD. In addition, FibroMeter VCTE had good diagnostic accuracy, similar to TE, for predicting severe fibrosis in autoimmune hepatitis (AIH) and performed even better in primary biliary cholangitis (PBC) as reported by Zachou et al. [191].

## Conclusions

Establishing an early diagnosis of liver fibrosis at early stages is essential to perform an accurate clinical intervention and prevent the progression from liver fibrosis to liver cirrhosis and hepatocellular carcinoma. Also, it is important to diagnose it as soon as possible as liver fibrosis is associated with long-term overall mortality, liver transplantation, and liver-related events [192]. Serum biomarkers are a very good option since they allow continuous monitoring, and they are less invasive compared to liver biopsy. All these non-invasive scoring systems, including direct and indirect serum biomarkers, yield high sensitivity but poor specificity, suggesting that they are best applied to exclude subjects without advanced fibrosis in MASLD populations [81], thereby avoiding unnecessary liver biopsies but not being useful to establish an accurate diagnosis by themselves. The use of these serum biomarkers and indirect indexes in a first step, or a combination of serum biomarkers with specific index such as ELF or combined with FibroScan or TE, are reliable strategies well recommended in clinical guidelines to better perform the liver fibrosis staging in different etiologies patients [16], as is a cost-effective diagnosis process. Furthermore, some of these biomarkers such as FIB-4 or NFS are considered an easy and economic tool that if implemented could cause a high impact in catching patients with liver fibrosis at the initial stages, where this pathology could be reverted [193, 194]. Although all this non invasive biomarkers and indexes have been study in at-risk population, they could be use as screening in groups of patients in primary care, such as type 2 diabetes mellitus, alcohol users disorders, metabolic risk factors or elevated liver enzymes, as there is still an extensive prevalence of chronic liver diseases in the general population [195]. For this reason, the American Diabetes Association (ADA) and the European Associations for the Study of Diabetes (EASD), of the Liver (EASL) and of Obesity (EASO) recommend screening for advanced liver fibrosis in all type 2 diabetes mellitus patients [81, 196]. Thus, every laboratory should evaluate and define the best diagnosis strategy in consensus with clinicians to rend the highest diagnostic performance in their target population.

## References

[1] Rinella ME, Neuschwander-Tetri BA, Siddiqui MS, Abdelmalek MF, Caldwell S, Barb D (2023). AASLD practice guidance on the clinical assessment and management of nonalcoholic fatty liver disease. Hepatology.

[2] Hernandez-Gea V, Friedman SL (2011). Pathogenesis of liver fibrosis. Annu Rev Pathol.

[3] Wiegand J, Berg T (2013). The etiology, diagnosis and prevention of liver cirrhosis: part 1 of a series on liver cirrhosis. Dtsch Arzteblatt Int.

[4] Kisseleva T (2017). The origin of fibrogenic myofibroblasts in fibrotic liver. Hepatology.

[5] Rockey DC, Bell PD, Hill JA (2015). Fibrosis--a common pathway to organ injury and failure. N Engl J Med.

[6] Ortiz C, Schierwagen R, Schaefer L, Klein S, Trepat X, Trebicka J (2021). Extracellular matrix remodeling in chronic liver disease. Curr Tissue Microenviron Rep.

[7] Iredale JP, Benyon RC, Pickering J, McCullen M, Northrop M, Pawley S (1998). Mechanisms of spontaneous resolution of rat liver fibrosis. Hepatic stellate cell apoptosis and reduced hepatic expression of metalloproteinase inhibitors. J Clin Invest.

[8] Rodimova S, Mozherov A, Elagin V, Karabut M, Shchechkin I, Kozlov D (2023). Effect of hepatic pathology on liver regeneration: the main metabolic mechanisms causing impaired hepatic regeneration. Int J Mol Sci.

[9] Reeves HL, Friedman SL (2002). Activation of hepatic stellate cells--a key issue in liver fibrosis. Front Biosci J Virtual Libr.

[10] Elpek GÖ (2014). Cellular and molecular mechanisms in the pathogenesis of liver fibrosis: an update. World J Gastroenterol.

[11] Zhou W-C, Zhang Q-B, Qiao L (2014). Pathogenesis of liver cirrhosis. World J Gastroenterol.

[12] Higashi T, Friedman SL, Hoshida Y (2017). Hepatic stellate cells as key target in liver fibrosis. Adv Drug Deliv Rev.

[13] Duarte S, Baber J, Fujii T, Coito AJ (2015). Matrix metalloproteinases in liver injury, repair and fibrosis. Matrix Biol.

[14] Moreira RK (2007). Hepatic stellate cells and liver fibrosis. Arch Pathol Lab Med.

[15] Lee JH, Joo I, Kang TW, Paik YH, Sinn DH, Ha SY (2020). Deep learning with ultrasonography: automated classification of liver fibrosis using a deep convolutional neural network. Eur Radiol.

[16] Castera L, Friedrich-Rust M, Loomba R (2019). Noninvasive assessment of liver disease in patients with nonalcoholic fatty liver disease. Gastroenterology.

[17] Chin JL, Pavlides M, Moolla A, Ryan JD (2016). Non-invasive markers of liver fibrosis: adjuncts or alternatives to liver biopsy?. Front Pharmacol.

[18] Sorrentino P, Tarantino G, Conca P, Perrella A, Terracciano ML, Vecchione R (2004). Silent non-alcoholic fatty liver disease-a clinical-histological study. J Hepatol.

[19] De Ritis F, Coltorti M, Giusti G (1957). An enzymic test for the diagnosis of viral hepatitis: the transaminase serum activities. Clin Chim Acta.

[20] Mofrad P, Contos MJ, Haque M, Sargeant C, Fisher RA, Luketic VA (2003). Clinical and histologic spectrum of nonalcoholic fatty liver disease associated with normal ALT values. Hepatology.

[21] Fracanzani AL, Valenti L, Bugianesi E, Andreoletti M, Colli A, Vanni E (2008). Risk of severe liver disease in nonalcoholic fatty liver disease with normal aminotransferase levels: a role for insulin resistance and diabetes. Hepatology.

[22] Williams ALB, Hoofnagle JH (1988). Ratio of serum aspartate to alanine aminotransferase in chronic hepatitis relationship to cirrhosis. Gastroenterology.

[23] Sheth SG, Flamm SL, Gordon FD, Chopra S (1998). AST/ALT ratio predicts cirrhosis in patients with chronic hepatitis C virus infection. Am J Gastroenterol.

[24] Nyblom H, Berggren U, Balldin J, Olsson R (2004). High AST/ALT ratio may indicate advanced alcoholic liver disease rather than heavy drinking. Alcohol Alcohol.

[25] Sorbi D, Boynton J, Lindor KD (1999). The ratio of aspartate aminotransferase to alanine aminotransferase: potential value in differentiating nonalcoholic steatohepatitis from alcoholic liver disease. Am J Gastroenterol.

[26] Nyblom H, Björnsson E, Simrén M, Aldenborg F, Almer S, Olsson R (2006). The AST/ALT ratio as an indicator of cirrhosis in patients with PBC. Liver Int.

[27] Guéchot J, Boisson RC, Zarski J-P, Sturm N, Calès P, Lasnier E (2013). AST/ALT ratio is not an index of liver fibrosis in chronic hepatitis C when aminotransferase activities are determinate according to the international recommendations. Clin Res Hepatol Gastroenterol.

[28] Wai C-T, Greenson JK, Fontana RJ, Kalbfleisch JD, Marrero JA, Conjeevaram HS (2003). A simple noninvasive index can predict both significant fibrosis and cirrhosis in patients with chronic hepatitis C. Hepatology.

[29] Tsochatzis EA, Crossan C, Longworth L, Gurusamy K, Rodriguez-Peralvarez M, Mantzoukis K (2014). Cost-effectiveness of noninvasive liver fibrosis tests for treatment decisions in patients with chronic hepatitis C. Hepatol Baltim.

[30] Martin J, Khatri G, Gopal P, Singal AG (2015). Accuracy of ultrasound and noninvasive markers of fibrosis to identify patients with cirrhosis. Dig Dis Sci.

[31] Usluer G, Erben N, Aykin N, Dagli O, Aydogdu O, Barut S (2012). Comparison of non-invasive fibrosis markers and classical liver biopsy in chronic hepatitis C. Eur J Clin Microbiol Infect Dis.

[32] Huang C, Seah JJ, Tan CK, Kam JW, Tan J, Teo EK (2021). Modified AST to platelet ratio index improves APRI and better predicts advanced fibrosis and liver cirrhosis in patients with non-alcoholic fatty liver disease. Clin Res Hepatol Gastroenterol.

[33] Zhao Y, Thurairajah PH, Kumar R, Tan J, Teo EK, Hsiang JC (2019). Novel non-invasive score to predict cirrhosis in the era of hepatitis C elimination: a population study of ex-substance users in Singapore. Hepatobiliary Pancreat Dis Int.

[34] Harrison SA, Oliver D, Arnold HL, Gogia S, Neuschwander-Tetri BA (2008). Development and validation of a simple NAFLD clinical scoring system for identifying patients without advanced disease. Gut.

[35] Park J, Kim G, Kim B-S, Han K-D, Kwon SY, Park SH (2022). The associations of hepatic steatosis and fibrosis using fatty liver index and BARD score with cardiovascular outcomes and mortality in patients with new-onset type 2 diabetes: a nationwide cohort study. Cardiovasc Diabetol.

[36] Forns X, Ampurdanès S, Llovet JM, Aponte J, Quintó L, Martínez-Bauer E (2002). Identification of chronic hepatitis C patients without hepatic fibrosis by a simple predictive model. Hepatol Baltim.

[37] Nabi O, Lacombe K, Boursier J, Mathurin P, Zins M, Serfaty L (2020). Prevalence and risk factors of nonalcoholic fatty liver disease and advanced fibrosis in general population: the French nationwide NASH-CO study. Gastroenterology.

[38] Romero Gómez M, Ramírez Martín del Campo M, Otero MA, Vallejo M, Corpas R, Castellano-Megías VM (2005). Comparative study of two models that use biochemical parameters for the non-invasive diagnosis of fibrosis in patients with hepatitis C. Med Clin.

[39] Hagström H, Talbäck M, Andreasson A, Walldius G, Hammar N (2020). Ability of noninvasive scoring systems to identify individuals in the population at risk for severe liver disease. Gastroenterology.

[40] Cusi K, Isaacs S, Barb D, Basu R, Caprio S, Garvey WT (2022). American association of clinical endocrinology clinical practice guideline for the diagnosis and management of nonalcoholic fatty liver disease in primary care and endocrinology clinical settings: co-sponsored by the American association for the study of liver diseases (AASLD). Endocr Pract.

[41] Anstee QM, Lawitz EJ, Alkhouri N, Wong VW-S, Romero-Gomez M, Okanoue T (2019). Noninvasive tests accurately identify advanced fibrosis due to NASH: baseline data from the STELLAR trials. Hepatology.

[42] Srivastava A, Gailer R, Tanwar S, Trembling P, Parkes J, Rodger A (2019). Prospective evaluation of a primary care referral pathway for patients with non-alcoholic fatty liver disease. J Hepatol.

[43] Itakura J, Kurosaki M, Setoyama H, Simakami T, Oza N, Korenaga M (2021). Applicability of APRI and FIB-4 as a transition indicator of liver fibrosis in patients with chronic viral hepatitis. J Gastroenterol.

[44] Xu X-Y, Wang W-S, Zhang Q-M, Li J-L, Sun J-B, Qin T-T (2019). Performance of common imaging techniques vs serum biomarkers in assessing fibrosis in patients with chronic hepatitis B: a systematic review and meta-analysis. World J Clin Cases.

[45] Xu X-Y, Kong H, Song R-X, Zhai Y-H, Wu X-F, Ai W-S (2014). The effectiveness of noninvasive biomarkers to predict hepatitis B-related significant fibrosis and cirrhosis: a systematic review and meta-analysis of diagnostic test accuracy. PLoS One.

[46] Xiao G, Yang J, Yan L (2015). Comparison of diagnostic accuracy of aspartate aminotransferase to platelet ratio index and fibrosis-4 index for detecting liver fibrosis in adult patients with chronic hepatitis B virus infection: a systemic review and meta-analysis. Hepatology.

[47] Sterling RK, Lissen E, Clumeck N, Sola R, Correa MC, Montaner J (2006). Development of a simple noninvasive index to predict significant fibrosis in patients with HIV/HCV coinfection. Hepatology.

[48] Kim BK, Kim DY, Park JY, Ahn SH, Chon CY, Kim JK (2010). Validation of FIB-4 and comparison with other simple noninvasive indices for predicting liver fibrosis and cirrhosis in hepatitis B virus-infected patients. Liver Int.

[49] Kim BK, Kim SA, Park YN, Cheong JY, Kim HS, Park JY (2007). Noninvasive models to predict liver cirrhosis in patients with chronic hepatitis B. Liver Int.

[50] Poynard T, Bedossa P (1997). Age and platelet count: a simple index for predicting the presence of histological lesions in patients with antibodies to hepatitis C virus. METAVIR and CLINIVIR Cooperative Study Groups. J Viral Hepat.

[51] Bedossa P, Poynard T (1996). An algorithm for the grading of activity in chronic hepatitis C. The METAVIR Cooperative Study Group. Hepatology.

[52] Group TFMCS, Bedossa P (1994). Intraobserver and interobserver variations in liver biopsy interpretation in patients with chronic hepatitis C. Hepatology.

[53] Dittrich M, Milde S, Dinkel E, Baumann W, Weitzel D (1983). Sonographic biometry of liver and spleen size in childhood. Pediatr Radiol.

[54] Batts KP, Ludwig J (1995). Chronic hepatitis. An update on terminology and reporting. Am J Surg Pathol.

[55] Roh YH, Kang B-K, Jun DW, Lee C, Kim M (2021). Role of FIB-4 for reassessment of hepatic fibrosis burden in referral center. Sci Rep.

[56] Moolla A, Motohashi K, Marjot T, Shard A, Ainsworth M, Gray A (2019). A multidisciplinary approach to the management of NAFLD is associated with improvement in markers of liver and cardio-metabolic health. Frontline Gastroenterol.

[57] McPherson S, Hardy T, Dufour J-F, Petta S, Romero-Gomez M, Allison M (2017). Age as a confounding factor for the accurate non-invasive diagnosis of advanced NAFLD fibrosis. Am J Gastroenterol.

[58] Canivet CM, Costentin C, Irvine KM, Delamarre A, Lannes A, Sturm N (2023). Validation of the new 2021 EASL algorithm for the noninvasive diagnosis of advanced fibrosis in NAFLD. Hepatology.

[59] Hagström H, Talbäck M, Andreasson A, Walldius G, Hammar N (2020). Repeated FIB-4 measurements can help identify individuals at risk of severe liver disease. J Hepatol.

[60] McPherson S, Stewart SF, Henderson E, Burt AD, Day CP (2010). Simple non-invasive fibrosis scoring systems can reliably exclude advanced fibrosis in patients with non-alcoholic fatty liver disease. Gut.

[61] Degos F, Perez P, Roche B, Mahmoudi A, Asselineau J, Voitot H (2010). Diagnostic accuracy of FibroScan and comparison to liver fibrosis biomarkers in chronic viral hepatitis: a multicenter prospective study (the FIBROSTIC study). J Hepatol.

[62] Madsen BS, Thiele M, Detlefsen S, Kjærgaard M, Møller LS, Trebicka J (2021). PRO-C3 and ADAPT algorithm accurately identify patients with advanced fibrosis due to alcohol-related liver disease. Aliment Pharmacol Ther.

[63] Angulo P, Hui JM, Marchesini G, Bugianesi E, George J, Farrell GC (2007). The NAFLD fibrosis score: a noninvasive system that identifies liver fibrosis in patients with NAFLD. Hepatology.

[64] Wei M, Xu Z, Pan X, Zhang X, Liu L, Yang B (2019). Serum GP73 – an additional biochemical marker for liver inflammation in chronic HBV infected patients with normal or slightly raised ALT. Sci Rep.

[65] Ampuero J, Pais R, Aller R, Gallego-Durán R, Crespo J, García-Monzón C (2020). Development and validation of Hepamet fibrosis scoring system-A simple, noninvasive test to identify patients with nonalcoholic fatty liver disease with advanced fibrosis. Clin Gastroenterol Hepatol.

[66] Benlloch S, Berenguer M, Prieto M, Rayón JM, Aguilera V, Berenguer J (2005). Prediction of fibrosis in HCV-infected liver transplant recipients with a simple noninvasive index. Liver Transpl.

[67] Xie S-B, Yao J-L, Zheng R-Q, Peng X-M, Gao Z-L (2003). Serum hyaluronic acid, procollagen type III and IV in histological diagnosis of liver fibrosis. Hepatobiliary Pancreat Dis Int.

[68] Nielsen MJ, Veidal SS, Karsdal MA, Ørsnes-Leeming DJ, Vainer B, Gardner SD (2015). Plasma Pro-C3 (N-terminal type III collagen propeptide) predicts fibrosis progression in patients with chronic hepatitis C. Liver Int.

[69] Daniels SJ, Leeming DJ, Eslam M, Hashem AM, Nielsen MJ, Krag A (2019). ADAPT: an algorithm incorporating PRO-C3 accurately identifies patients with NAFLD and advanced fibrosis. Hepatology.

[70] Kumagai E, Mano Y, Yoshio S, Shoji H, Sugiyama M, Korenaga M (2016). Serum YKL-40 as a marker of liver fibrosis in patients with non-alcoholic fatty liver disease. Sci Rep.

[71] Tran A, Benzaken S, Saint-Paul M-C, Guzman-Granier E, Hastier P, Pradier C (2000). Chondrex (YKL-40), a potential new serum fibrosis marker in patients with alcoholic liver disease. Eur J Gastroenterol Hepatol.

[72] Jiang Z, Wang S, Jin J, Ying S, Chen Z, Zhu D (2020). The clinical significance of serum chitinase 3-like 1 in hepatitis B–related chronic liver diseases. J Clin Lab Anal.

[73] Saitou Y, Shiraki K, Yamanaka Y, Yamaguchi Y, Kawakita T, Yamamoto N (2005). Noninvasive estimation of liver fibrosis and response to interferon therapy by a serum fibrogenesis marker, YKL-40, in patients with HCV-associated liver disease. World J Gastroenterol.

[74] Mak L-Y, Wong DK-H, Cheung K-S, Seto W-K, Lai C-L, Yuen M-F (2018). Role of serum M2BPGi levels on diagnosing significant liver fibrosis and cirrhosis in treated patients with chronic hepatitis B virus infection. Clin Transl Gastroenterol.

[75] Hur M, Park M, Moon H-W, Choe WH, Lee CH (2022). Comparison of non-invasive clinical algorithms for liver fibrosis in patients with chronic hepatitis B to reduce the need for liver biopsy: application of enhanced liver fibrosis and mac-2 binding protein glycosylation isomer. Ann Lab Med.

[76] Parkes J, Roderick P, Harris S, Day C, Mutimer D, Collier J (2010). Enhanced liver fibrosis test can predict clinical outcomes in patients with chronic liver disease. Gut.

[77] Day J, Patel P, Parkes J, Rosenberg W (2019). Derivation and performance of standardized enhanced liver fibrosis (ELF) test thresholds for the detection and prognosis of liver fibrosis. J Appl Lab Med.

[78] Wong GL-H, Chan HL-Y, Choi PC-L, Chan AW-H, Yu Z, Lai JW-Y (2014). Non-invasive algorithm of enhanced liver fibrosis and liver stiffness measurement with transient elastography for advanced liver fibrosis in chronic hepatitis B. Aliment Pharmacol Ther.

[79] Becker L, Salameh W, Sferruzza A, Zhang K, ng Chen R, Malik R (2009). Validation of hepascore, compared with simple indices of fibrosis, in patients with chronic hepatitis C virus infection in United States. Clin Gastroenterol Hepatol.

[80] Adams LA, Bulsara M, Rossi E, DeBoer B, Speers D, George J (2005). Hepascore: an accurate validated predictor of liver fibrosis in chronic hepatitis C infection. Clin Chem.

[81] European Association for the Study of the Liver (EASL), European Association for the Study of Diabetes (EASD), European Association for the Study of Obesity (EASO) (2016). EASL–EASD–EASO clinical practice guidelines for the management of non-alcoholic fatty liver disease. J Hepatol.

[82] Graupera I, Thiele M, Serra-Burriel M, Caballeria L, Roulot D, Wong GL-H (2022). Low accuracy of FIB-4 and NAFLD fibrosis scores for screening for liver fibrosis in the population. Clin Gastroenterol Hepatol.

[83] Munteanu M, Tiniakos D, Anstee Q, Charlotte F, Marchesini G, Bugianesi E (2016). Diagnostic performance of FibroTest, SteatoTest and ActiTest in patients with NAFLD using the SAF score as histological reference. Aliment Pharmacol Ther.

[84] Salkic NN, Jovanovic P, Hauser G, Brcic M (2014). FibroTest/fibrosure for significant liver fibrosis and cirrhosis in chronic hepatitis B: a meta-analysis. Off J Am Coll Gastroenterol.

[85] Naveau S, Gaudé G, Asnacios A, Agostini H, Abella A, Barri-Ova N (2009). Diagnostic and prognostic values of noninvasive biomarkers of fibrosis in patients with alcoholic liver disease. Hepatology.

[86] Vali Y, Lee J, Boursier J, Spijker R, Verheij J, Brosnan MJ (2021). FibroTest for evaluating fibrosis in non-alcoholic fatty liver disease patients: a systematic review and meta-analysis. J Clin Med.

[87] Munteanu M, Pais R, Peta V, Deckmyn O, Moussalli J, Ngo Y (2018). Long-term prognostic value of the FibroTest in patients with non-alcoholic fatty liver disease, compared to chronic hepatitis C, B, and alcoholic liver disease. Aliment Pharmacol Ther.

[88] Yao M, Wang L, Leung PSC, Li Y, Liu S, Wang L (2018). The clinical significance of GP73 in immunologically mediated chronic liver diseases: experimental data and literature review. Clin Rev Allergy Immunol.

[89] Yang S-L, Zeng C, Fang X, He Q-J, Liu L-P, Bao S-Y (2018). Hepatitis B virus upregulates GP73 expression by activating the HIF-2α signaling pathway. Oncol Lett.

[90] Xu Z, Liu L, Pan X, Wei K, Wei M, Liu L (2015). Serum Golgi protein 73 (GP73) is a diagnostic and prognostic marker of chronic HBV liver disease. Medicine.

[91] Xu Z, Shen J, Pan X, Wei M, Liu L, Wei K (2018). Predictive value of serum Golgi protein 73 for prominent hepatic necroinflammation in chronic HBV infection. J Med Virol.

[92] Marrero JA, Romano PR, Nikolaeva O, Steel L, Mehta A, Fimmel CJ (2005). GP73, a resident Golgi glycoprotein, is a novel serum marker for hepatocellular carcinoma. J Hepatol.

[93] Gatselis NK, Tornai T, Shums Z, Zachou K, Saitis A, Gabeta S (2020). Golgi protein-73: a biomarker for assessing cirrhosis and prognosis of liver disease patients. World J Gastroenterol.

[94] Ke M-Y, Wu X-N, Zhang Y, Wang S, Lv Y, Dong J (2019). Serum GP73 predicts posthepatectomy outcomes in patients with hepatocellular carcinoma. J Transl Med.

[95] Li Y, Yang Y, Li Y, Zhang P, Ge G, Jin J (2021). Use of GP73 in the diagnosis of non-alcoholic steatohepatitis and the staging of hepatic fibrosis. J Int Med Res.

[96] Cao Z, Li Z, Wang H, Liu Y, Xu Y, Mo R (2017). Algorithm of Golgi protein 73 and liver stiffness accurately diagnoses significant fibrosis in chronic HBV infection. Liver Int.

[97] Cao Z, Li Z, Wang Y, Liu Y, Mo R, Ren P (2017). Assessment of serum Golgi protein 73 as a biomarker for the diagnosis of significant fibrosis in patients with chronic HBV infection. J Viral Hepat.

[98] Rigor J, Diegues A, Presa J, Barata P, Martins-Mendes D (2022). Noninvasive fibrosis tools in NAFLD: validation of APRI, BARD, FIB-4, NAFLD fibrosis score, and Hepamet fibrosis score in a Portuguese population. Postgrad Med.

[99] Zambrano-Huailla R, Guedes L, Stefano JT, de Souza AAA, Marciano S, Yvamoto E (2020). Diagnostic performance of three non-invasive fibrosis scores (Hepamet, FIB-4, NAFLD fibrosis score) in NAFLD patients from a mixed Latin American population. Ann Hepatol.

[100] Higuera-de-la-Tijera F, Córdova-Gallardo J, Buganza-Torio E, Barranco-Fragoso B, Torre A, Parraguirre-Martínez S (2021). Hepamet fibrosis score in nonalcoholic fatty liver disease patients in Mexico: lower than expected positive predictive value. Dig Dis Sci.

[101] Tafur Sánchez CN, Durá Gil M, Alemán Domínguez Del Río A, Hernández Pérez CM, Mora Cuadrado N, de la Cuesta SG (2022). The practical utility of non-invasive indices in metabolic hepatic steatosis. Endocrinol Diabetes Nutr.

[102] Younes R, Caviglia GP, Govaere O, Rosso C, Armandi A, Sanavia T (2021). Long-term outcomes and predictive ability of non-invasive scoring systems in patients with non-alcoholic fatty liver disease. J Hepatol.

[103] Ampuero J, Aller R, Gallego-Durán R, Crespo J, Calleja JL, García-Monzón C (2020). Significant fibrosis predicts new-onset diabetes mellitus and arterial hypertension in patients with NASH. J Hepatol.

[104] Alonso C, Fernández-Ramos D, Varela-Rey M, Martínez-Arranz I, Navasa N, Van Liempd SM (2017). Metabolomic identification of subtypes of nonalcoholic steatohepatitis. Gastroenterology.

[105] Cantero I, Elorz M, Abete I, Marin BA, Herrero JI, Monreal JI (2019). Ultrasound/elastography techniques, lipidomic and blood markers compared to magnetic resonance imaging in non-alcoholic fatty liver disease adults. Int J Med Sci.

[106] Mayo R, Crespo J, Martínez‐Arranz I, Banales JM, Arias M, Mincholé I (2018). Metabolomic-based noninvasive serum test to diagnose nonalcoholic steatohepatitis: results from discovery and validation cohorts. Hepatol Commun.

[107] Bril F, Millán L, Kalavalapalli S, McPhaul MJ, Caulfield MP, Martinez-Arranz I (2018). Use of a metabolomic approach to non-invasively diagnose non-alcoholic fatty liver disease in patients with type 2 diabetes mellitus. Diabetes Obes Metab.

[108] Rostami S, Parsian H (2013). Hyaluronic acid: from biochemical characteristics to its clinical translation in assessment of liver fibrosis. Hepatitis Mon.

[109] Gudowska M, Cylwik B, Chrostek L (2017). The role of serum hyaluronic acid determination in the diagnosis of liver diseases. Acta Biochim Pol.

[110] Stickel F, Poeschl G, Schuppan D, Conradt C, Strenge-Hesse A, Fuchs FS (2003). Serum hyaluronate correlates with histological progression in alcoholic liver disease. Eur J Gastroenterol Hepatol.

[111] Mizuno M, Shima T, Oya H, Mitsumoto Y, Mizuno C, Isoda S (2017). Classification of patients with non-alcoholic fatty liver disease using rapid immunoassay of serum type IV collagen compared with liver histology and other fibrosis markers. Hepatol Res.

[112] Sowa J-P, Heider D, Bechmann LP, Gerken G, Hoffmann D, Canbay A (2013). Novel algorithm for non-invasive assessment of fibrosis in NAFLD. PLoS One.

[113] Chwist A, Hartleb M, Lekstan A, Kukla M, Gutkowski K, Kajor M (2014). A composite model including visfatin, tissue polypeptide-specific antigen, hyaluronic acid, and hematological variables for the diagnosis of moderate-to-severe fibrosis in nonalcoholic fatty liver disease: a preliminary study. Pol Arch Med Wewn.

[114] Halfon P, Bourlière M, Pénaranda G, Deydier R, Renou C, Botta-Fridlund D (2005). Accuracy of hyaluronic acid level for predicting liver fibrosis stages in patients with hepatitis C virus. Comp Hepatol.

[115] Rossi E, Adams LA, Bulsara M, Jeffrey GP (2007). Assessing liver fibrosis with serum marker models. Clin Biochem Rev.

[116] El Serafy MA, Kassem AM, Omar H, Mahfouz MS, El Said El Raziky M (2017). APRI test and hyaluronic acid as non-invasive diagnostic tools for post HCV liver fibrosis: systematic review and meta-analysis. Arab J Gastroenterol.

[117] Geramizadeh B, Janfeshan K, Saberfiroozi M (2008). Serum hyaluronic acid as a noninvasive marker of hepatic fibrosis in chronic hepatitis B. Saudi J Gastroenterol.

[118] Peters L, Mocroft A, Soriano V, Rockstroh J, Rauch A, Karlsson A (2013). Hyaluronic acid levels predict risk of hepatic encephalopathy and liver-related death in HIV/viral hepatitis coinfected patients. PLoS One.

[119] Nunes D, Fleming C, Offner G, O’Brien M, Tumilty S, Fix O (2005). HIV infection does not affect the performance of noninvasive markers of fibrosis for the diagnosis of hepatitis C virus-related liver disease. J Acquired Immune Defic Syndr.

[120] Neuman MG, Cohen LB, Nanau RM (2016). Hyaluronic acid as a non-invasive biomarker of liver fibrosis. Clin Biochem.

[121] Bril F, Leeming DJ, Karsdal MA, Kalavalapalli S, Barb D, Lai J (2019). Use of plasma fragments of propeptides of type III, V, and VI procollagen for the detection of liver fibrosis in type 2 diabetes. Diabetes Care.

[122] Annoni G, Colombo M, Cantaluppi MC, Khlat B, Lampertico P, Rojkind M (1989). Serum Type III procollagen peptide and laminin (Lam-P1) detect alcoholic hepatitis in chronic alcohol abusers. Hepatology.

[123] Gabrielli GB, Capra F, Casaril M, Squarzoni S, Tognella P, Dagradi R (1997). Serum laminin and type III procollagen in chronic hepatitis C. Diagnostic value in the assessment of disease activity and fibrosis. Clin Chim Acta.

[124] Guéchot J, Laudat A, Loria A, Serfaty L, Poupon R, Giboudeau J (1996). Diagnostic accuracy of hyaluronan and type III procollagen amino-terminal peptide serum assays as markers of liver fibrosis in chronic viral hepatitis C evaluated by ROC curve analysis. Clin Chem.

[125] Mosca A, Comparcola D, Romito I, Mantovani A, Nobili V, Byrne CD (2019). Plasma N-terminal propeptide of type III procollagen accurately predicts liver fibrosis severity in children with non-alcoholic fatty liver disease. Liver Int.

[126] Mak KM, Mei R (2017). Basement membrane type IV collagen and laminin: an overview of their biology and value as fibrosis biomarkers of liver disease. Anat Rec.

[127] Walsh KM, Fletcher A, MacSween RN, Morris AJ (2000). Basement membrane peptides as markers of liver disease in chronic hepatitis C. J Hepatol.

[128] Misaki M, Shima T, Yano Y, Sumita Y, Kano U, Murata T (1990). Basement membrane-related and type III procollagen-related antigens in serum of patients with chronic viral liver disease. Clin Chem.

[129] Stefano JT, Guedes LV, de Souza AAA, Vanni DS, Alves VAF, Carrilho FJ (2021). Usefulness of collagen type IV in the detection of significant liver fibrosis in nonalcoholic fatty liver disease. Ann Hepatol.

[130] Praktiknjo M, Lehmann J, Nielsen MJ, Schierwagen R, Uschner FE, Meyer C (2018). Acute decompensation boosts hepatic collagen type III deposition and deteriorates experimental and human cirrhosis. Hepatol Commun.

[131] Karsdal MA, Henriksen K, Nielsen MJ, Byrjalsen I, Leeming DJ, Gardner S (2016). Fibrogenesis assessed by serological type III collagen formation identifies patients with progressive liver fibrosis and responders to a potential antifibrotic therapy. Am J Physiol Gastrointest Liver Physiol.

[132] Karsdal MA, Hjuler ST, Luo Y, Rasmussen DGK, Nielsen MJ, Holm Nielsen S (2019). Assessment of liver fibrosis progression and regression by a serological collagen turnover profile. Am J Physiol Gastrointest Liver Physiol.

[133] Yilmaz Y, Eren F (2019). Serum biomarkers of fibrosis and extracellular matrix remodeling in patients with nonalcoholic fatty liver disease: association with liver histology. Eur J Gastroenterol Hepatol.

[134] Livzan MA, Lapteva IV, Krolevets TS (2016). Assessment of matrix metalloproteinases and their tissue inhibitors for non-invasive diagnosis of liver fibrosis in patients with nonalcoholic fatty liver disease. Exp Clin Gastroenterol.

[135] Boeker KHW, Haberkorn CI, Michels D, Flemming P, Manns MP, Lichtinghagen R (2002). Diagnostic potential of circulating TIMP-1 and MMP-2 as markers of liver fibrosis in patients with chronic hepatitis C. Clin Chim Acta.

[136] Kasahara A, Hayashi N, Mochizuki K, Oshita M, Katayama K, Kato M (1997). Circulating matrix metalloproteinase-2 and tissue inhibitor of metalloproteinase-1 as serum markers of fibrosis in patients with chronic hepatitis C: relationship to interferon response. J Hepatol.

[137] Munsterman ID, Kendall TJ, Khelil N, Popa M, Lomme R, Drenth JPH (2018). Extracellular matrix components indicate remodelling activity in different fibrosis stages of human non-alcoholic fatty liver disease. Histopathology.

[138] Irvine KM, Okano S, Patel PJ, Horsfall LU, Williams S, Russell A (2021). Serum matrix metalloproteinase 7 (MMP7) is a biomarker of fibrosis in patients with non-alcoholic fatty liver disease. Sci Rep.

[139] Wang S, Hu M, Qian Y, Jiang Z, Shen L, Fu L (2020). CHI3L1 in the pathophysiology and diagnosis of liver diseases. Biomed Pharmacother.

[140] Huang H, Wu T, Mao J, Fang Y, Zhang J, Wu L (2015). CHI3L1 is a liver-enriched, noninvasive biomarker that can be used to stage and diagnose substantial hepatic fibrosis. OMICS J Integr Biol.

[141] Yan L, Deng Y, Zhou J, Zhao H, Wang G, Zhang D-Z (2018). Serum YKL-40 as a biomarker for liver fibrosis in chronic hepatitis B patients with normal and mildly elevated ALT. Infection.

[142] Shirabe K, Bekki Y, Gantumur D, Araki K, Ishii N, Kuno A (2018). Mac-2 binding protein glycan isomer (M2BPGi) is a new serum biomarker for assessing liver fibrosis: more than a biomarker of liver fibrosis. J Gastroenterol.

[143] Kuno A, Ikehara Y, Tanaka Y, Ito K, Matsuda A, Sekiya S (2013). A serum ‘sweet-doughnut’ protein facilitates fibrosis evaluation and therapy assessment in patients with viral hepatitis. Sci Rep.

[144] Toshima T, Shirabe K, Ikegami T, Yoshizumi T, Kuno A, Togayachi A (2015). A novel serum marker, glycosylated Wisteria floribunda agglutinin-positive Mac-2 binding protein (WFA(+)-M2BP), for assessing liver fibrosis. J Gastroenterol.

[145] Xu H, Kong W, Liu L, Chi X, Wang X, Wu R (2017). Accuracy of M2BPGi, compared with Fibro Scan^®^, in analysis of liver fibrosis in patients with hepatitis C. BMC Gastroenterol.

[146] Nakamura M, Kanda T, Jiang X, Haga Y, Takahashi K, Wu S (2017). Serum microRNA-122 and Wisteria floribunda agglutinin-positive Mac-2 binding protein are useful tools for liquid biopsy of the patients with hepatitis B virus and advanced liver fibrosis. PLoS One.

[147] Nishikawa H, Enomoto H, Iwata Y, Hasegawa K, Nakano C, Takata R (2016). Clinical significance of serum Wisteria floribunda agglutinin positive Mac-2-binding protein level and high-sensitivity C-reactive protein concentration in autoimmune hepatitis. Hepatol Res.

[148] Nishikawa H, Enomoto H, Iwata Y, Kishino K, Shimono Y, Hasegawa K (2016). Clinical significance of serum Wisteria floribunda agglutinin positive Mac-2-binding protein level in non-alcoholic steatohepatitis. Hepatol Res.

[149] Lai L-L, Chan W-K, Sthaneshwar P, Nik Mustapha NR, Goh K-L, Mahadeva S (2017). Serum Wisteria floribunda agglutinin-positive Mac-2 binding protein in non-alcoholic fatty liver disease. PLoS One.

[150] Yamada N, Sanada Y, Tashiro M, Hirata Y, Okada N, Ihara Y (2017). Serum Mac-2 binding protein glycosylation isomer predicts grade F4 liver fibrosis in patients with biliary atresia. J Gastroenterol.

[151] Nishikawa H, Enomoto H, Iwata Y, Hasegawa K, Nakano C, Takata R (2016). Impact of serum Wisteria floribunda agglutinin positive Mac-2-binding protein and serum interferon-γ-inducible protein-10 in primary biliary cirrhosis. Hepatol Res.

[152] Umetsu S, Inui A, Sogo T, Komatsu H, Fujisawa T (2018). Usefulness of serum Wisteria floribunda agglutinin-positive Mac-2 binding protein in children with primary sclerosing cholangitis. Hepatol Res.

[153] Hanai T, Shiraki M, Ohnishi S, Miyazaki T, Ideta T, Kochi T (2015). Impact of serum glycosylated Wisteria floribunda agglutinin positive Mac-2 binding protein levels on liver functional reserves and mortality in patients with liver cirrhosis. Hepatol Res.

[154] Tsuji Y, Namisaki T, Kaji K, Takaya H, Nakanishi K, Sato S (2020). Comparison of serum fibrosis biomarkers for diagnosing significant liver fibrosis in patients with chronic hepatitis B. Exp Ther Med.

[155] Nagata H, Nakagawa M, Asahina Y, Sato A, Asano Y, Tsunoda T (2017). Effect of interferon-based and -free therapy on early occurrence and recurrence of hepatocellular carcinoma in chronic hepatitis C. J Hepatol.

[156] Zou X, Zhu M-Y, Yu D-M, Li W, Zhang D-H, Lu F-J (2017). Serum WFA+ -M2BP levels for evaluation of early stages of liver fibrosis in patients with chronic hepatitis B virus infection. Liver Int.

[157] Ishii A, Nishikawa H, Enomoto H, Iwata Y, Kishino K, Shimono Y (2017). Clinical implications of serum Wisteria floribunda agglutinin-positive Mac-2-binding protein in treatment-naïve chronic hepatitis B. Hepatol Res.

[158] Ura K, Furusyo N, Ogawa E, Hayashi T, Mukae H, Shimizu M (2016). Serum WFA(+) -M2BP is a non-invasive liver fibrosis marker that can predict the efficacy of direct-acting anti-viral-based triple therapy for chronic hepatitis C. Aliment Pharmacol Ther.

[159] Suda T, Okawa O, Masaoka R, Gyotoku Y, Tokutomi N, Katayama Y (2017). Shear wave elastography in hepatitis C patients before and after antiviral therapy. World J Hepatol.

[160] Shinkai N, Nojima M, Iio E, Matsunami K, Toyoda H, Murakami S (2018). High levels of serum Mac-2-binding protein glycosylation isomer (M2BPGi) predict the development of hepatocellular carcinoma in hepatitis B patients treated with nucleot(s)ide analogues. J Gastroenterol.

[161] Saleh SA, Salama MM, Alhusseini MM, Mohamed GA (2020). M2BPGi for assessing liver fibrosis in patients with hepatitis C treated with direct-acting antivirals. World J Gastroenterol.

[162] Rosenberg WMC, Voelker M, Thiel R, Becka M, Burt A, Schuppan D (2004). Serum markers detect the presence of liver fibrosis: a cohort study. Gastroenterology.

[163] Sharma C, Cococcia S, Ellis N, Parkes J, Rosenberg W (2021). Systematic review: accuracy of the enhanced liver fibrosis test for diagnosing advanced liver fibrosis and cirrhosis. J Gastroenterol Hepatol.

[164] Day JW, Rosenberg WM (2018). The enhanced liver fibrosis (ELF) test in diagnosis and management of liver fibrosis. Br J Hosp Med.

[165] Rasmussen DN, Thiele M, Johansen S, Kjærgaard M, Lindvig KP, Israelsen M (2021). Prognostic performance of 7 biomarkers compared to liver biopsy in early alcohol-related liver disease. J Hepatol.

[166] Vali Y, Lee J, Boursier J, Spijker R, Löffler J, Verheij J (2020). Enhanced liver fibrosis test for the non-invasive diagnosis of fibrosis in patients with NAFLD: a systematic review and meta-analysis. J Hepatol.

[167] Mayo MJ, Parkes J, Adams-Huet B, Combes B, Mills AS, Markin RS (2008). Prediction of clinical outcomes in primary biliary cirrhosis by serum enhanced liver fibrosis assay. Hepatology.

[168] Martinez SM, Fernández-Varo G, González P, Sampson E, Bruguera M, Navasa M (2011). Assessment of liver fibrosis before and after antiviral therapy by different serum marker panels in patients with chronic hepatitis C. Aliment Pharmacol Ther.

[169] Trépo E, Potthoff A, Pradat P, Bakshi R, Young B, Lagier R (2011). Role of a cirrhosis risk score for the early prediction of fibrosis progression in hepatitis C patients with minimal liver disease. J Hepatol.

[170] Guha IN, Parkes J, Roderick P, Chattopadhyay D, Cross R, Harris S (2008). Noninvasive markers of fibrosis in nonalcoholic fatty liver disease: validating the European liver fibrosis panel and exploring simple markers. Hepatology.

[171] Crespo G, Gambato M, Millán O, Casals G, Ruiz P, Londoño MC (2016). Early non-invasive selection of patients at high risk of severe hepatitis C recurrence after liver transplantation. Transpl Infect Dis.

[172] Younossi ZM, Felix S, Jeffers T, Younossi E, Nader F, Pham H (2021). Performance of the enhanced liver fibrosis test to estimate advanced fibrosis among patients with nonalcoholic fatty liver disease. JAMA Netw Open.

[173] Lichtinghagen R, Pietsch D, Bantel H, Manns MP, Brand K, Bahr MJ (2013). The enhanced liver fibrosis (ELF) score: normal values, influence factors and proposed cut-off values. J Hepatol.

[174] Huang Y, Adams LA, Joseph J, Bulsara MK, Jeffrey GP (2017). The ability of Hepascore to predict liver fibrosis in chronic liver disease: a meta-analysis. Liver Int.

[175] Anty R, Vanbiervliet G, Gelsi E, Rosenthal A, Huet PM, Saint-Paul MC (2006). CA 17-Évaluation externe des scores sanguins de fibrose hépatique (fibrometre, hepascore, apri) au cours des hépatopathies alcooliques. Gastroentérol Clin Biol.

[176] Chrostek L, Przekop D, Gruszewska E, Gudowska-Sawczuk M, Cylwik B (2019). Noninvasive indirect markers of liver fibrosis in alcoholics. Biomed Res Int.

[177] Adams LA, George J, Bugianesi E, Rossi E, De Boer WB, van der Poorten D (2011). Complex non-invasive fibrosis models are more accurate than simple models in non-alcoholic fatty liver disease. J Gastroenterol Hepatol.

[178] Wang Z, Bertot LC, Jeffrey GP, Joseph J, Garas G, de Boer B (2022). Serum fibrosis tests guide prognosis in metabolic dysfunction–associated fatty liver disease patients referred from primary care. Clin Gastroenterol Hepatol.

[179] Rossi E, Adams LA, Ching HL, Bulsara M, MacQuillan GC, Jeffrey GP (2013). High biological variation of serum hyaluronic acid and Hepascore, a biochemical marker model for the prediction of liver fibrosis. Clin Chem Lab Med.

[180] Calès P, Boursier J, Oberti F, Hubert I, Gallois Y, Rousselet M-C (2008). FibroMeters: a family of blood tests for liver fibrosis. Gastroentérol Clin Biol.

[181] Leroy V, Sturm N, Faure P, Trocme C, Marlu A, Hilleret M-N (2014). Prospective evaluation of FibroTest^®^, FibroMeter^®^, and HepaScore^®^ for staging liver fibrosis in chronic hepatitis B: comparison with hepatitis C. J Hepatol.

[182] Boursier J, Vergniol J, Guillet A, Hiriart J-B, Lannes A, Le Bail B (2016). Diagnostic accuracy and prognostic significance of blood fibrosis tests and liver stiffness measurement by FibroScan in non-alcoholic fatty liver disease. J Hepatol.

[183] Leroy V, Halfon P, Bacq Y, Boursier J, Rousselet MC, Bourlière M (2008). Diagnostic accuracy, reproducibility and robustness of fibrosis blood tests in chronic hepatitis C: a meta-analysis with individual data. Clin Biochem.

[184] Boursier J, Bacq Y, Halfon P, Leroy V, de Ledinghen V, de Muret A (2009). Improved diagnostic accuracy of blood tests for severe fibrosis and cirrhosis in chronic hepatitis C. Eur J Gastroenterol Hepatol.

[185] Calès P, Lainé F, Boursier J, Deugnier Y, Moal V, Oberti F (2009). Comparison of blood tests for liver fibrosis specific or not to NAFLD. J Hepatol.

[186] Calès P, Oberti F, Michalak S, Hubert-Fouchard I, Rousselet M-C, Konaté A (2005). A novel panel of blood markers to assess the degree of liver fibrosis. Hepatology.

[187] Guillaume M, Moal V, Delabaudiere C, Zuberbuhler F, Robic M-A, Lannes A (2019). Direct comparison of the specialised blood fibrosis tests FibroMeterV2G and Enhanced Liver Fibrosis score in patients with non-alcoholic fatty liver disease from tertiary care centres. Aliment Pharmacol Ther.

[188] Ducancelle A, Leroy V, Vergniol J, Sturm N, Le Bail B, Zarski JP (2017). A single test combining blood markers and elastography is more accurate than other fibrosis tests in the main causes of chronic liver diseases. J Clin Gastroenterol.

[189] Van Dijk A-M, Vali Y, Mak AL, Lee J, Tushuizen ME, Zafarmand MH (2021). Systematic review with meta-analyses: diagnostic accuracy of FibroMeter tests in patients with non-alcoholic fatty liver disease. J Clin Med.

[190] Dincses E, Yilmaz Y (2015). Diagnostic usefulness of FibroMeter VCTE for hepatic fibrosis in patients with nonalcoholic fatty liver disease. Eur J Gastroenterol Hepatol.

[191] Zachou K, Lygoura V, Arvaniti P, Giannoulis G, Gatselis NK, Koukoulis GK (2021). FibroMeter scores for the assessment of liver fibrosis in patients with autoimmune liver diseases. Ann Hepatol.

[192] Angulo P, Kleiner DE, Dam-Larsen S, Adams LA, Bjornsson ES, Charatcharoenwitthaya P (2015). Liver fibrosis, but no other histologic features, is associated with long-term outcomes of patients with nonalcoholic fatty liver disease. Gastroenterology.

[193] Srivastava A, Jong S, Gola A, Gailer R, Morgan S, Sennett K (2019). Cost-comparison analysis of FIB-4, ELF and fibroscan in community pathways for non-alcoholic fatty liver disease. BMC Gastroenterol.

[194] Crossan C, Majumdar A, Srivastava A, Thorburn D, Rosenberg W, Pinzani M (2019). Referral pathways for patients with NAFLD based on non-invasive fibrosis tests: diagnostic accuracy and cost analysis. Liver Int.

[195] Canivet CM, Boursier J (2022). Screening for liver fibrosis in the general population: where do we stand in 2022?. Diagnostics.

[196] American Diabetes Association (2020). 4. Comprehensive medical evaluation and assessment of comorbidities: standards of medical care in diabetes-2020. Diabetes Care.

